# Recent Progresses of Polarons: Fundamentals and Roles in Photocatalysis and Photoelectrocatalysis

**DOI:** 10.1002/advs.202305139

**Published:** 2023-11-10

**Authors:** Zhizhen Ren, Zhijian Shi, Haifeng Feng, Zhongfei Xu, Weichang Hao

**Affiliations:** ^1^ School of Physics Beihang University Beijing 100191 China; ^2^ College of Environmental Science and Engineering North China Electric Power University Beijing 102206 China

**Keywords:** catalysts, photocatalysis, photoelectrocatalysis, polarons

## Abstract

Photocatalysis and photoelectrocatalysis are promising ways in the utilization of solar energy. To address the low efficiency of photocatalysts and photoelectrodes, in‐depth understanding of their catalytic mechanism is in urgent need. Recently, polaron is considered as an influential factor in catalysis, which brings researchers a new approach to modify photocatalysts and photoelectrodes. In this review, brief introduction of polaron is given first, followed by which models and recent experimentally observations of polarons are reviewed. Studies about roles of polarons in photocatalysis and photoelectrocatalysis are listed in order to provide some inspiration in exploring the mechanism and improving the efficiency of photocatalysis and photoelectrocatalysis.

## Graphical abstract

Polaron can play an influential role in photocatalysis and photoelectrocatalysis. In this review, brief introduction of polaron, followed with models and recent experimentally observations of polarons are reviewed. Several studies about roles of polarons in photocatalysis and photoelectrocatalysis are then listed to provide some inspiration in exploring the mechanism and improving the efficiency of photocatalysis and photoelectrocatalysis.

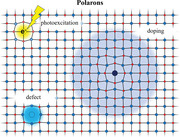

## 1 Introduction

Photocatalysis and photoelectrocatalysis are promising catalytic methods in green and sustainable energy utilization and chemical synthesis, especially for utilizing solar energy to deal with environmental pollution, CO_2_ fixation, water splitting, and so on.^[^ ^1^, ^2^, ^3^, ^4^, ^5^, ^6^ ^]^ In photocatalysis and photoelectrocatalysis, chemical reactions can be activated or accelerated by the reactive charge carriers generated in photocatalysts or photoelectrodes under light irradiations. However, it should be realized that these two methods are far from large‐scale application, mainly because of low efficiency, poor stability, and high cost of photocatalysts and photoelectrodes.^[^ ^7^ ^]^ Thus, in last several decades, great efforts have been made in these two fields aiming at creating catalytic candidates with high efficiency, selectivity, stability, and affordable costs.

In the light of the complexity of photocatalysis and photoelectrocatalysis, many materials have been designed and tested as catalysts with a lot of mechanisms and strategies proposed in the scientific studies. In brief, light absorption, separation, and transfer of photoexcited charge carriers, surface active sites, molecular adsorption/desorption are key factors affecting the performance of a certain catalyst,^[^ ^1^ ^]^ which are determined by the physical and chemical properties of this material. Considering that the above key factors are actually physical or chemical processes happen at atomic scale or molecular scale, in‐depth understanding of the structure‐activity relationship and relating properties at nano‐ and micro‐scale of these catalysts are necessary to reveal how catalytic processes take place in these materials, particularly on surfaces.

In recent several years, an important factor, polaron, is brought into researchers’ consideration and getting more and more attentions in photocatalysis and photoelectrocatalysis. A polaron is a quasi‐particle formed by an excess charge and accompanying deformations of surrounding lattice.^[^ ^8^, ^9^, ^10^, ^11^, ^12^, ^13^, ^14^, ^15^, ^16^ ^]^ Polarons can easily form in polar semiconductors, ionic crystals, organic semiconductors, hybrid perovskites, and so on, many of which are well‐known photocatalysts or photoelectrodes, such as TiO_2_,^[^ ^17^, ^18^, ^19^, ^20^ ^]^ SrTiO_3_,^[^ ^21^ ^]^ Fe_2_O_3_,^[^ ^22^ ^]^ and BiVO_4_.^[^ ^23^, ^24^, ^25^ ^]^ Once polarons are introduced in photocatalysts and photoelectrodes by doping, surface functionalization or photoexcitation, many properties including light absorption, charge separation and transfer, surface active sites, molecular adsorption/desorption, and local electronic properties are expected to be affected.^[^ ^26^, ^27^, ^28^, ^29^ ^]^ Consequently, the effects of polaron on the performances of photocatalysts and photoelectrodes cannot be neglected. Thanks to the developments of characterization methods, material control methods, computational simulation, etc. a bunch of new experimental and theoretic progresses have been made in a few materials such as metal oxides,^[^ ^30^, ^31^ ^]^ perovskite,^[^ ^21^ ^]^ two‐dimensional (2D) atomic crystals,^[^ ^32^, ^33^, ^34^, ^35^, ^36^ ^]^ and so on.^[^ ^16^ ^]^ The idea of modifying or promoting the performances of photocatalysts and photoelectrodes through tailoring the polaron reacting properties is being stimulated in the research field.

In this review, we will briefly introduce the polaron and its theoretic models, and then summarize experimental works in recent years of characterizing polarons and their roles in photocatalysis and photoelectrocatalysis. We hope this review could provide readers with understanding of polarons and, more importantly, with inspirations in catalytic mechanism and control methods at the microscopic scale.

## 2 Models of Polaron

**Figure 1:**
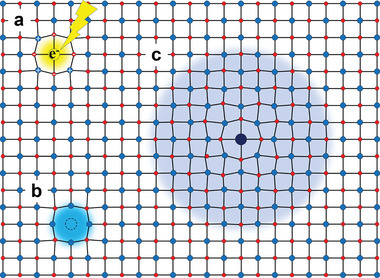
Diagram of a polaron: a) A Holstein polaron (small polaron) formed by a photoexcited electron. b) A Holstein polaron induced by lattice defect. c) A Fröhlich polaron (large polaron) induced by a doped ion.

Landau studied the electron trapping behavior in crystal lattice in 1933,^[^ ^8^ ^]^ and the concept of polaron was first proposed by Pekar in 1946 to describe a quasi‐particle formed by an excess charge carrier (electron or hole) with its surrounding ions displaced in ionic or polar crystals.^[^ ^9^, ^16^, ^37^ ^]^ When an excess electron (hole) is generated in a crystal, Coulomb interaction between the electron (hole) and surrounding ions will pull or push away these ions from their initial positions and form a localized polarization field. This excess charge and the distortion of surrounding ions together is called a polaron. In field theory, the interaction between charge and surrounding ions can be described by a cloud of virtual phonons. In this sense, the polaron is a quasi‐particle composed of a charge carrier and its surrounding virtual phonon cloud. An excess charge carrier can be generated through many methods such as doping, surface functionalization, and photoexcitation. Actually, polarons exist directly in ionic crystals as the charge carrier, which had been pointed out by Landau and Pekar.^[^ ^10^, ^11^ ^]^ An early polaron model described by Pekar is Landau‐Pekar model.^[^ ^9^ ^]^ In this model, the ionic crystal itself is regarded as a continuous dielectric. More accurate models were proposed later in 1950 and 1959 by Fröhlich^[^ ^12^, ^13^ ^]^ and Holstein^[^ ^14^, ^15^ ^]^ to describe two main types of polaron, that are Fröhlich polaron (large polaron) and Holstein polaron (small polaron), respectively (Diagrams in **Figure** **1**). Till now, polaron types have been extended to more types, such as bipolaron,^[^ ^38^ ^]^ magnetic polaron,^[^ ^39^, ^40^ ^]^ Jahn‐Teller polaron,^[^ ^41^, ^42^ ^]^ etc.

Fröhlich polaron and Holstein polaron are classified by their size, which is mainly determined by the range of electron‐phonon coupling (EPC). For a Fröhlich polaron, its size and EPC range are much larger than the lattice constant. While for a Holstein polaron, its size is restricted to the lattice constant with the excess charge carrier trapped by polarization field.

Although the size of a polaron can be affected by a lot of factors, there are still some methods to estimate the size of a polaron. It has been mentioned above that a polaron can be described as a quasi‐particle composed of a charge carrier and its surrounding virtual phonons cloud. In this point of view, the lattice distortion caused by the excess charge carrier is determined by the interaction between charge carrier and virtual phonon. The size of a polaron can be roughly estimated by the position uncertainty of the charge carrier under interaction with longitudinal optical (LO) virtual phonon.^[^ ^43^ ^]^ Considering that an electron absorbs and emits a LO virtual phonon in a polaron, the electron has an energy uncertainty

(1) $$\begin{array}{c} \Delta E\;=\;\hbar \omega_{L} \end{array}$$

where ω_*L*_ and ℏω_*L*_ are the frequency and the energy of a LO phonon. The energy of an electron takes the following form

(2) $$\begin{array}{c} E\;=\frac{\hbar^{2}k^{2}}{2m_{e}} \end{array}$$

where *m_e_* is the effective mass of electron. Uncertainty of wave vector is

(3) $$\begin{array}{c} \Delta k\;={\left(\frac{2m_{e}\omega_{L}}{\hbar }\right)}^{\frac{1}{2}} \end{array}$$

and finally, the position uncertainty of the electron is

(4) $$\begin{array}{c} \Delta r\;=\frac{1}{\Delta k}\;={\left(\frac{\hbar }{2m_{e}\omega_{L}}\right)}^{\frac{1}{2}} \end{array}$$

In a polar semiconductor, the effective mass of band electron is small. As a result, Δ*r* is much larger than lattice constant, in which case the Fröhlich polaron often forms. By contrast, in most ionic crystals, the band electron has a relatively large effective mass and a strong electron‐phonon interaction. Thus, the position uncertainty of an excess charge carrier, Δ*r*, is close to the lattice constant, in which case the polaron formed is so called Holstein polaron.

The early model given by Pekar describes an excess electron in dielectric polarizable continuum.^[^ ^9^ ^]^ The equation takes the following form:^[^ ^44^ ^]^

(5) $$\begin{array}{c} \left(-\frac{\nabla^{2}}{2m}-e\int dr^{\prime }P\left(r^{\prime }\right)\cdot \nabla^{\prime }\frac{1}{\left|r^{\prime }-r\right|}\right)\psi \left(r\right)=E_{0}\psi \left(r\right) \end{array}$$

The effective mass of polaron can be calculated from:

(6) $$\begin{array}{c} m^{*}\approx 0.02\alpha^{4}m \end{array}$$

where α is EPC constant:

(7) $$\begin{array}{c} \alpha \;=\frac{e^{2}}{\kappa }\;\sqrt{\frac{m}{2\omega_{0}}},\;\frac{1}{\kappa }=\frac{1}{\varepsilon }\;-\frac{1}{\varepsilon_{0}} \end{array}$$

The larger α is, the more significant the polaronic effect is, resulting in a smaller polaron size. However, Pekar's model is actually not a valid model.^[^ ^44^ ^]^ Under continuum approximation, the lattice discreteness is ignored, which is usually applied for large polaron. At the same time, this model requires a large coupling constant α to solve. But for large polaron, EPC effect is usually weak, showing small coupling constant α. As a result, Pekar's model is not applicable for either large polaron or small polaron.

To describe polaron more accurately, Fröhlich and Holstein had proposed two models for large and small polaron, known as Fröhlich polaron and Holstein polaron, respectively.

### 2.1 Fröhlich Polaron (Large Polaron)

The model for a Fröhlich polaron also follows continuum approximation by using the interaction between electron and lattice polarization field to replace electron‐phonon interaction. It describes polarons with large size and weak EPC.^[^ ^44^ ^]^ Fröhlich Hamiltonian has the form:

(8) $$\begin{array}{c} H\;=\;-\frac{\nabla^{2}}{2m}+\sum_{q}\omega_{q}\left(a_{q}^{+}a_{q}+\frac{1}{2}\right)+\sum_{q}\left(V_{q}a_{q}e^{iq\cdot r}+h.c.\right) \end{array}$$

where the first term is the kinetic energy of an electron with effective mass *m*. The second term is the Hamiltonian of phonons with $a_{q}^{+}$ and *a_q_* being the creation and annihilation operators for LO phonons. The last term describes the interaction potential for an electron in the lattice polarization field.

Under weak EPC, energy and effective mass of Fröhlich polaron can be calculated through perturbation theory at T = 0 K:

(9) $$\begin{array}{c} \;E_{k}=\;-\alpha \hbar \omega_{L}+\frac{\hbar^{2}k^{2}}{2m^{*}} \end{array}$$

(10) $$\begin{array}{c} \;m^{*}=\frac{m}{1-\frac{\alpha }{6}}\;\approx m\left(1+\frac{\alpha }{6}\right) \end{array}$$

(11) $$\begin{array}{c} \;V_{q}=\;i\frac{\hbar \omega_{L}}{q}{\left(\frac{\hbar }{2m\omega_{L}}\right)}^{\frac{1}{4}}{\left(4\pi \alpha \right)}^{\frac{1}{2}} \end{array}$$

(12) $$\begin{array}{c} \alpha \;=\frac{e^{2}}{2\hbar \omega_{L}}\;{\left(\frac{2m\omega_{L}}{\hbar }\right)}^{\frac{1}{2}}\left(\frac{1}{\varepsilon_{\infty }}-\frac{1}{\varepsilon_{0}}\right) \end{array}$$

In Fröhlich Hamiltonian, *V_q_* are Fourier components of the electron‐phonon interaction. For *V_q_*, it was assumed that:^[^ ^45^ ^]^ (1) the spatial extension of the polaron is large compared with the lattice parameters of the solid (“continuum” approximation); (2) spin and relativistic effects can be neglected; (3) the band electron has parabolic dispersion; and (4) in conjunction with the first approximation it is also assumed that LO phonons of interest for the interaction are long‐wavelength phonons with constant frequency ω_*L*_. In real crystals, values of coupling constant α in Fröhlich model are ≈0.02–0.2 in III‐V materials, 0.3–0.5 in II‐VI materials, and 2–5 in alkali halides.^[^ ^45^ ^]^ For different value of α and intensity of EPC, different theories and methods were proposed to get accurate solution of Fröhlich Hamiltonian. In weak coupling regime, α < 1, perturbation theory was applied to solve Fröhlich Hamiltonian.^[^ ^12^, ^13^ ^]^

However, for a larger α, perturbation theory becomes invalid. Lee, Low, and Pines had proposed Lee–Low–Pines (LLP) canonical transformation to solve the model with α < 6 in 1953.^[^ ^46^ ^]^ In 1955, Feynman developed an all‐coupling theory for continuum Fröhlich polaron using path‐integral.^[^ ^44^, ^47^ ^]^ This theory could be applied in almost any regime of α and give a more accurate solution in α ranging 3 to 11 than LLP theory.

As pointed out by Landau and Pekar that polarons could exist directly in ionic crystals as charge carriers. The mobility of polarons has a great impact on charge transport in materials.^[^ ^48^, ^49^ ^]^ For Fröhlich polarons, EPC is relatively weak. Electrons (or holes) are not localized by surrounding ions and polarized field. The mobility of a Fröhlich polaron is more like a free charge carrier with larger effective mass. Boltzmann equation^[^ ^50^, ^51^ ^]^ and the Feynman, Hellwarth, Iddings, and Platzman (FHIP) approach^[^ ^52^, ^53^, ^54^ ^]^ have been adopted to theoretical described Fröhlich polarons in ionic crystal.

### 2.2 Holstein Polaron (Small Polaron)

Holstein polaron refers to a polaron whose size is equal to or smaller than lattice constant. Being aware of this, a model describing this kind of polaron must take the interaction between the electron and its surrounding atoms into account. A simple Holstein model involves two vibrating molecules (1 and 2) and an electron hopping between them.^[^ ^43^ ^]^ Hamiltonian of this model has the form:

(13) $$\begin{array}{c} H\;=t\left(c_{1}^{+}c_{2}+c_{2}^{+}c_{1}\right)+H_{ph}\;+H_{e-ph} \end{array}$$

(14) $$\begin{array}{c} \;H_{ph}=\;-\frac{1}{2M}\frac{\partial^{2}}{\partial x^{2}}+\frac{kx^{2}}{2} \end{array}$$

(15) $$\begin{array}{c} \;H_{e-ph}=\;fx\left(c_{1}^{+}c_{1}+c_{2}^{+}c_{2}\right) \end{array}$$

where *H_ph_* is the vibration part, *H*_*e* − *ph*_ represent for electron‐phonon interaction.

A Holstein polaron, which could be regarded as a trapped carrier in a potential well, is not as free as a Fröhlich polaron. Its motion in lattice is more like a hopping process. Marcus theory was proposed in studying its hopping mobility,^[^ ^55^, ^56^ ^]^ which had been further developed by Emin, Holstein, Austin, and Mott (EHAM) later.^[^ ^57^, ^58^, ^59^ ^]^ Hopping motion of a Holstein polaron can be considered as nonadiabatic or adiabatic. In the non‐adiabatic regime, polarized ionic distortion, or the ion vibrations are faster than the electron hopping, meaning that hopping frequency of electrons is smaller than phonon frequency.^[^ ^44^, ^48^, ^60^ ^]^ In the contrary, electron moves faster than polarized ions in the adiabatic regime.^[^ ^48^, ^61^, ^62^ ^]^ Adiabatic approximation assumes the position of atom nuclei is fixed, while non‐adiabatic method takes the scattering effect by phonons into consideration, which is depended on temperature.^[^ ^22^ ^]^ Hopping studies by first‐principles molecular dynamic (FPMD) can refer to some papers.^[^ ^16^, ^17^, ^29^, ^63^, ^64^ ^]^

## 3 Experimental Observation

### 3.1 Scanning Tunneling Microscopy

Scanning tunneling microscope (STM) can obtain surface information including morphologies, electrical properties, and so on by analyzing tunneling current between STM probe and sample surface. STM has been widely used in studying surface properties of materials. Benefiting from its atomic resolution and the ability to detect electronic states, STM can be used to identify surface defect, reconstruction, charge accumulation, and other characteristics that could directly or indirectly represent polarons on the surface.

**Figure 2:**
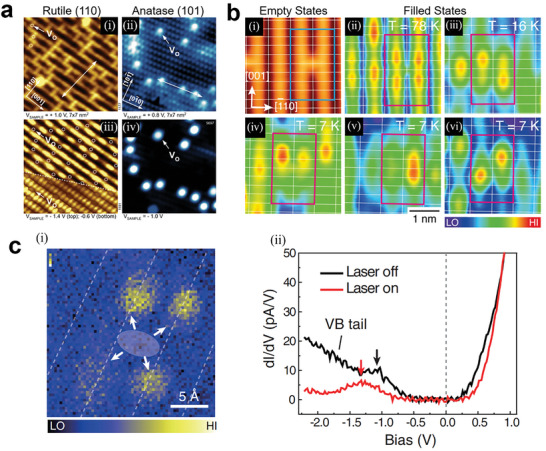
a) STM images of surface local electronic structure of rutile (110) (left) at T = 78 K and anatase (101) (right) at T = 6 K. (i), (ii) empty states, and (iii), (iv) filled states were obtained under constant‐height scan. Oxygen vacancies (V_O_s) are marked with arrows and circles. Reproduced with permission.^[^ ^17^ ^]^ Copyright 2014, American Physical Society. b) STM images of isolated V_O_s on rutile (110). (i) empty states and (ii)‐(vi) filled states were obtained under different temperature: (i), (ii) 78 K, (iii) 16 K, and (iv)‐(vi) 7 K. Reproduced with permission.^[^ ^18^ ^]^ Copyright 2016, American Physical Society. c) dI/dV spectra of polarons. (i) The dI/dV mapping of V_O_. The white dashed lines highlight the Ti_5c_ rows and the white arrows indicate the distribution of the polaron states. Set points of STM and dI/dV mapping: *V* = 1.7 V and *I* = 70 pA. The dI/dV mapping was acquired with an open feedback loop at the bias: V = −1.2 V. (ii) Red (black) curves are dI/dV spectra of polaron states on rutile (110) with (without) 700 nm laser irradiation. The arrows mark the peak positions of polaron states. Reproduced with permission.^[^ ^19^ ^]^ Copyright 2020, American Physical Society.

TiO_2_ is a typical transition metal oxide semiconductor that has been exhaustively studied. The formation of polarons on rutile and anatase TiO_2_ results in different characteristics due to their different crystalline structures.^[^ ^17^, ^65^, ^66^, ^67^, ^68^, ^69^, ^70^ ^]^ Setvin et al. had studied excess electrons in rutile (110) and anatase (101) using STM, spectroscopy, and density function theory (DFT).^[^ ^17^ ^]^ As shown in **Figure** **2**a, local electronic structures of oxygen vacancies (V_O_s) doped rutile (110) at *T* = 78 K (left) and anatase (101) at *T* = 6 K (right) were obtained by STM. (i) and (ii) of Figure 2a are empty states images of rutile (110) and anatase (101), respectively, which show the spatial positions of V_O_s as marked with arrows and circles. (iii) and (iv) of Figure 2a are the corresponding filled states images that show the distributions of electronic states introduced by V_O_s. On rutile (110), filled local density of states (LDOS) [Figure 2a(iii)] is mainly from surface Ti_5c_ atoms and is small at V_O_s, which is thought to be determined by the position of polarons. Their calculations showed that small polarons on rutile (110), which are formed by excess electrons introduced by V_O_s, could position on several Ti sites at surface or subsurface near the V_O_s. However, on anatase (101), the additional electronic states pinned right on the two surface Ti sites at a V_O_s, showing separate LDOS signal position at V_O_s in filled states image [Figure 2a(iv)].

**Figure 3:**
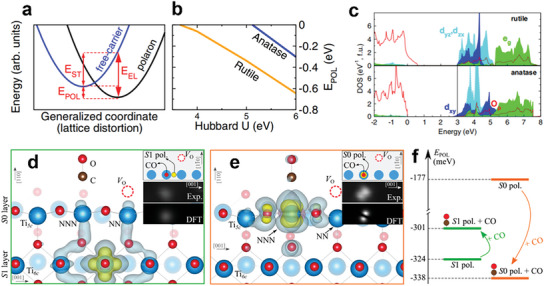
a) configuration coordinate diagram showing the polaronic (*E*_POL_), lattice (*E*_ST_), and electronic (*E_EL_*) energies as a function of lattice distortion for the polaronic and delocalized solution. b) *E*_POL_ as a function of Hubbard U in bulk rutile (orange) and anatase (blue). c) orbitally decomposed density of states (DOS) in rutile and anatase. Reproduced with permission.^[^ ^17^ ^]^ Copyright 2014, American Physical Society. d,e) electronic charge density of the surface d) and subsurface e) polarons in presence of CO. The insets represent the experimental and simulated filled state STM images. f) Formation energy of surface and subsurface polarons on rutile (110) with and without adsorbed CO. Reproduced with permission.^[^ ^26^ ^]^ Copyright 2019, American Physical Society.

DFT calculation was used to explain why two types of polaron form in two crystalline phases of TiO_2_ in this work by Setvin et al.^[^ ^17^ ^]^ The formation energy of the polaron represents the sum of the two parts:

(16) $$\begin{array}{c} \;E_{\mathrm{POL}}=E_{\mathrm{ST}}\;+E_{EL} \end{array}$$

the first part *E*_ST_ is the energy cost needed to distort the lattice to bind the polaron, and the second part is the obtained energy *E_EL_* by localizing electrons at the distorted lattice position. The magnitude of the formation energy is a significant factor in determining the stability and locality of a polaron,^[^ ^28^, ^71^ ^]^ as shown in **Figure** **3**a.^[^ ^17^ ^]^ DFT+U method was used to study polarons in TiO_2_, as shown in Figure 3b. In different crystalline phases of TiO_2_, Hubbard U values used will affect the formation energy of polarons. Figure 3c demonstrates that the calculated conduction band (CB) of anatase is more expansive than that of rutile, and the conduction band minimum (CBM) is also shallower, indicating a stronger combination between the d_xy_ orbitals of neighboring titanium atoms in anatase. Therefore, the associated energy gain *E_EL_* of rutile is higher than that of anatase, and the polaron formation energy is lower. As a result, an excess electron tends to be trapped at a Ti site in rutile and form a small polaron, while in anatase, excess electrons act like free carriers.

On rutile (110), as excess electrons could position on several Ti sites at surface or subsurface near the V_O_s, the filled LDOS image in STM at *T* = 78 K is actually a weighted average of different electron distributions [Figure 2a(iii)]. Yim et al. studied polaron configurations around a V_O_ on rutile (110) by STM at different temperatures, as shown in Figure 2b.^[^ ^18^ ^]^ Empty states image [Figure 2b(i)] remains the same at different temperatures, but filled states shows different polaron distributions under different temperatures. These temperature‐dependent phenomena can be explained by the hopping motion of small polarons. At *T* = 78 K, electron distribution around a V_O_ presents a four‐lobe structure centering at the V_O_, indicating that the two polarons introduced by the V_O_ continuously hop among the several Ti sites around the V_O_. The hopping frequency is much faster than scan rate of STM at this temperature, which means the obtained electron distribution in the image is a weighted average of different configurations. With the temperature decreases, the hopping frequency of polarons also decreases, leading to the symmetry of the four‐lobe structure breaks and two of the lobes become brighter. At *T* = 7 K, as shown in Figure 2b(iv)–(vi), the distribution of electrons presents almost only two lobes with three pattens, indicating that the two polarons are almost pinned in one STM scan process.

Guo et al. had studied photoresponse of a polaron using scanning tunneling spectroscopy (STS). A dI/dV curve by STM on a polaron measured with and without a 700 nm laser irradiation is shown in Figure 2c.^[^ ^19^ ^]^ Figure 2c(i) shows the four‐lobe pattern of polaron distribution. In Figure 2c(ii), under irradiation, peak position of polaron states presents a downward shift, which is marked by arrows. Meanwhile, valence band (VB) tail state is suppressed, and the CB edge increases. The most possible cause of these changes is the photoinduced electronic transition between the polaron states and CB or VB. Calculations illustrated that the shift of polaron state is caused by the decrease of the on‐site Coulomb interaction energy when the electrons are excited out of the in‐gap states. The suppression of the VB tail and the increase of the CB edge are results of the depletion of the polaron states and the accumulation of free electrons in the CBM, respectively.

Two recent works about polarons in 2D CoCl_2_ by Liu et al.^[^ ^72^ ^]^ and Cai et al.^[^ ^73^ ^]^ had provided the possibility to create and control polarons by STM tip. In these two parallel works, polarons were found to form on CoCl_2_ monolayer on highly oriented pyrolytic graphite (HOPG) substrate. STM was used to observe, create, and control the polarons at low temperature. By applying a certain amount of positive or negative bias on sample, electrons could be injected from STM tip to sample or be removed from sample, which lead to the creation or annihilation of electron polarons on defect‐free lattice. In addition, different types of polarons (two by Liu et al., four by Cai et al.) located at different lattice sites were observed and the interconversion among different types of polarons can be realized by precisely regulating the applied bias.

Atomic force microscopy (AFM) is also a widely used microscopy technique in surface science. By detecting atomic interaction force, AFM can directly obtain surface information. Different with STM, AFM would not be affected by the conductivity of sample. Nowadays the highly developed non‐contact AFM (nc‐AFM) has atomic resolution comparable to STM. Polarons could also be indirectly observed near surface defects or through other characteristics by AFM.

### 3.2 Angle‐Resolved Photoemission Spectroscopy

**Figure 4:**
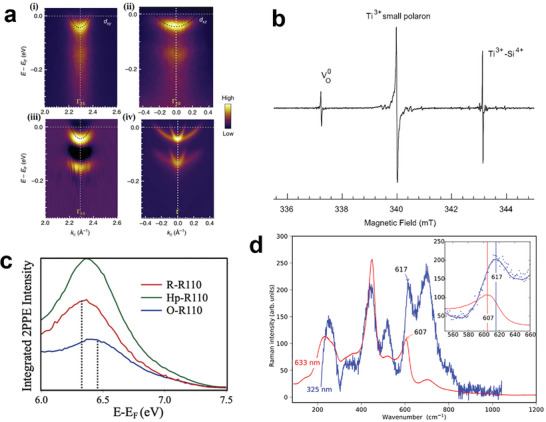
a) Observation of large polaron by ARPES at SrTiO_3_ surface. (i) Dispersion from Γ_11_along^[^ ^11^ ^]^ direction with d_xy_ orbital character in *k_x_* − *k_y_* space. (ii) Dispersion from Γ_10_ along [01] direction with d_xz_ orbital character. The momentum cuts are shown in dashed while lines. (iii) Energy‐second‐derivative image of spectra in (i). (iv) Simulated large polaron dispersions with d_xy_/d_yz_ and d_xz_ orbital characters. Reproduced with permission.^[^ ^21^ ^]^ Copyright 2015, Springer Nature. b) Photoinduced EPR spectrum from a rutile TiO_2_ crystal, taken at 15 K during exposure to 442 nm laser light. The signal in the middle is from the Ti^3+^ self‐trapped electron. Reproduced with permission.^[^ ^20^ ^]^ Copyright 2013, American Physical Society. c) 2PPE spectra from rutile R‐R110, H_p_‐R110, and O‐R110 (the three terminations were produced consecutively in situ), recorded with p‐polarized 3.54 eV (350 nm) light. Reproduced with permission.^[^ ^27^ ^]^ Copyright 2021, American Physical Society. d) Raman spectra of rutile taken with the 632.8 nm laser (red line) and the 325 nm UV laser (blue line) with ℏω = 3.8 *eV* > *E_gap_*. The inset shows a magnified view of the spectra around the 607 cm^−1^ and 617 cm^−1^ peaks. Reproduced with permission.^[^ ^74^ ^]^ Copyright 2017, American Physical Society.

Angle‐resolved photoemission spectroscopy (ARPES) is a technique to measure electron energies and momenta in materials based on photoelectric effect. Electronic band structure and Fermi surface can be mapped by measuring kinetic energies and corresponding emission angles. As mentioned above, the mobility of a large polaron in crystals is more like a free charge carrier with a larger effective mass. Thus, spectroscopies such as ARPES can be used to observe large polarons. Observation of 2D large polaron spectra by ARPES at SrTiO_3_ (001) surface is obtained by Chen et al., as shown in **Figure** **4**a.^[^ ^21^ ^]^ Electrons with different orbital symmetry were measured, d_xy_ in (i) and d_xz_ in (ii). It can be found that there are replica bands for each orbital, lying ≈90 meV higher than the original, which can be distinguished from the energy‐second‐derivative spectra in Figure 4a(iii) and corresponds well with simulation [Figure 4a(iv)]. These replicas are attributed to the many‐body effect, specifically, the EPC. The characteristic energy, ≈90 meV, corresponds to one surface polar LO phonon mode. This work showed that the existence of polarons can be reflected by replica bands measured in ARPES.

### 3.3 Electron Paramagnetic Resonance

Electron paramagnetic resonance (EPR) can be applied to measure unpaired electrons in materials, which is often used in studying metal complexes and organic radicals. The excess electron in crystal, which localized at a lattice ion and form a polaron, can be treated as an unpaired electron. Thus, EPR can be used to identify and characterize the intrinsic self‐trapped electrons. As shown in Figure 4b, a photoinduced EPR spectrum for rutile TiO_2_ crystal was obtained at 15 K during exposure to 442 nm laser light by Yang et al.^[^ ^20^ ^]^ In rutile TiO_2_, an electron could be excited by laser light and self‐trapped at a Ti^4+^ ion at a low temperature to form an intrinsic small polaron. Three clear signals appeared in EPR spectrum after irradiation. The signal at 337.2 mT is the low‐field component of the doublet assigned to the *S* = 1 neutral charge state of the V_O_. The signal at 343.1 mT has been tentatively assigned to the Ti^3+^‐Si^4+^ center. The signal at 340.0 mT represents the intrinsic small polaron at Ti^3+^. The self‐trapped electron can be only observed with low microwave power at temperatures near and below 20 K, as the trapped electrons would be thermally activated and recombine with holes at higher temperature.

### 3.4 Two‐Photon Photoelectron Spectroscopy

Two‐photon photoelectron (2PPE) spectroscopy is a technique for the study of electronic structure and electronic excitations at surfaces. Femtosecond to picosecond laser pulses are used to photoexcite electrons twice with a certain time interval. The kinetic energy and the emission angle of the photoelectron are measured under different time interval to investigate the population and relaxation pathways of photoexcitation. Tanner et al. used 2PPE to identify the photoexcitation of surface and bulk polarons in rutile (110), which involves the excitation of polaronic excess electrons in surface‐localized band‐gap states (BGS) to conduction band states, as shown in Figure 4c.^[^ ^27^ ^]^ Reduced rutile(110), denoted as R‐R110, was hydroxylated from residual water in the ultra‐high vaccum (UHV) chamber to form H_p_‐R110, after which 2PPE intensity increased. It had been assumed that this increase is caused by the stabilization of photoexcited electrons after hydroxylation. The 2PPE signal is a sum of contributions from polarons in the surface, subsurface, and bulk. Then BGS on surface and subsurface was removed after the sample was exposed to 54 Langmuir (L) of O_2_, which is denoted as O‐R110. On O‐R110 curve, only bulk polarons contributed to the spectrum, the resonance energy presents a ≈0.15 eV shift. This experimental result and the corresponding calculations showed the associated intermediate states of the bulk polarons lie at 0.14–0.20 eV higher energy than the surface.

### 3.5 Raman Spectroscopy

Raman spectroscopy measures the scattering spectrum between samples and the incident light based on Raman scattering effect. This technique is used to investigate the vibration and rotation of molecules, and can be used to analysis chemical structure, phase, molecule interaction, and so on. Incident laser in Raman spectroscopy could interact with molecule vibrations and phonons, thus the existence of polarons could be reflected in the spectrum. As shown in Figure 4d, in a work by Kolesov et al. Raman spectra of rutile were obtained under normal conditions (633 nm, red line) and after UV laser excitations (325 nm, blue line).^[^ ^74^ ^]^ The peak at 607 cm^−1^ in 633 nm spectrum corresponds for A_1g_ mode, and this peak shifts to the higher wave number of 617 cm^−1^ in 325 nm spectrum. This shift up of the A_1g_ mode is attributed to polaron‐induced structural changes. The stiffening of the A_1g_ mode was observed both in the experimental Raman spectra and in DFT simulations. They suggested that the polaron breaks the crystal symmetry and the original crystalline A_1g_ mode is transformed into a new oxygen breathing mode.

### 3.6 High Time Resolution Spectroscopies

Apart from above methods, some other high time resolution methods have also been used in studying the formation and dynamic of polarons.^[^ ^30^, ^31^, ^75^, ^76^, ^77^, ^78^ ^]^ Guzelturk et al. used femtosecond resolution diffuse X‐ray scattering measurement and observed a nanoscale structure distortion on picosecond timescales caused by charge carriers in lead halide perovskites (MA)PbBr_3_.^[^ ^30^ ^]^ When a charge is excited by light and forms a polaron, it would cause lattice distortion. In this work, Guzelturk et al. observed the expansion of the distortion, which is a few angstroms at first, and rapidly expands outward to a diameter of about 5 billionths of a meter, or ≈10 layers of atoms. In another work by Cinquanta et al. Ultrafast optical pump‐THz probe spectroscopy combining with DFT was used to demonstrate the presence of large polarons in the dielectric response of thin films of CsPbBr_3_ nanocrystal.^[^ ^31^ ^]^ Three phonon modes obtained by optical‐pump THz‐probe spectroscopy at a pump‐probe delay of 3 ps corresponded well with calculated phonon modes. Carrier dynamics were also studied through the frequency‐averaged dielectric response of the sample at different pump‐probe delay. The experimantal observation methods of polarons are summaried in **Table** **1**.

**Table 1:** Different experimental observation of polarons.

Technique	Material	Size	Type	Origin
STM	Rutile TiO_2_ (110)^[^ ^17^ ^]^	Small	Electron	Oxygen vacancy
	Anatase TiO_2_ (101)^[^ ^17^ ^]^	Large	Electron	Oxygen vacancy
	2D CoCl_2_^[^ ^72^, ^73^ ^]^	Small	Electron	Electron injection
ARPES	SrTiO_3_ (001)^[^ ^21^ ^]^	Large	Electron	Oxygen vacancy
EPR	Rutile TiO_2_^[^ ^20^ ^]^	Small	Electron	Photoexcitation
2PPE	Rutile TiO_2_^[^ ^27^ ^]^	Small	Electron	Photoexcitation
Raman	Rutile TiO_2_^[^ ^74^ ^]^	Small	Electron	Photoexcitation
Diffuse X‐ray scattering	(MA)PbBr_3_^[^ ^30^ ^]^	Large	*Unknown*	Photoexcitation
THz spectroscopy	CsPbBr_3_^[^ ^31^ ^]^	Large	Hole	Photoexcitation
	BiVO_4_^[^ ^23^ ^]^	Small	Hole	Photoexcitation
XUV	Fe_2_O_3_^[^ ^22^ ^]^	Small	Electron	Photoexcitation

## 4 Roles of Polarons in Catalyst Properties and Catalysis Processes

### 4.1 Adsorption of Molecules on the Surface

**Figure 5:**
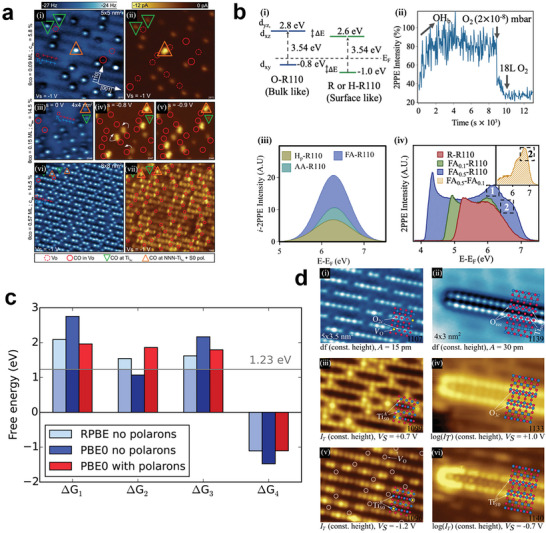
a) nc‐AFM (blue‐white) and filled state STM (yellow‐black) images of CO adsorption on the rutile (110) surfaces of different surface reduction degree. Reproduced with permission.^[^ ^26^ ^]^ Copyright 2019, American Physical Society. b) (i) schematic of polaron excitations in bulk and surface environments of rutile (110) on the basis of calculations and 2PPE spectra. (ii) time evolution of the rutile (110) 2PPE peak monitored over the course of 200 min. Reproduced with permission.^[^ ^27^ ^]^ Copyright 2021, American Physical Society. (iii) 2PPE spectra of the H_p_‐R110, FA‐R110, and AA‐R110 terminations at a photon energy of 3.54 eV (p‐[001], 350 nm). (iv) 2PPE spectra (hν = 3.54 eV, 350 nm, s‐[001]) of a reduced rutile (110) sample (R‐R110) taken continuously as formic acid is dosed in situ. The inset shows the spectrum with 0.5 ML coverage minus that at 0.1 ML coverage. Reproduced with permission.^[^ ^28^ ^]^ Copyright 2022, American Chemical Society. c) Gibbs free energy steps corresponding to the intermediates of the OER on rutile (110). The gray line indicates the standard potential of water splitting. Reproduced with permission.^[^ ^79^ ^]^ Copyright 2018, American Chemical Society. d) AFM and STM images of surface reconstruction on rutile TiO_2_, AFM images (i) and (ii) show the highest O atoms (O_2c_ and O_rec_) on the surface, and STM images show the empty (iii), (iv) and filled (v), (vi) states. Reproduced with permission.^[^ ^29^ ^]^ Copyright 2017, American Physical Society.

Adsorption of molecules on the surface of catalyst is one of the key steps in catalysis. The adsorption position and its stability could greatly affect catalytic efficiency. **Figure** **5**a shows a study of interaction between CO and polarons on rutile (110) by Reticcioli et al. Nc‐AFM and filled state STM were used to recognize the adsorption position of CO.^[^ ^26^ ^]^ Polarons with different concentration were obtained by different reduction degree of TiO_2_. On a slightly reduced surface (c_VO_ = 5.8%) with a low CO coverage [θ = 0.05 monolayer (ML)], as shown in Figure 5a(i),(ii), CO molecules are predominantly adsorbed on Ti_5c_ site (triangles), and few are adsorbed on V_O_s (solid circles), the latter cannot be observed by filled state STM because there is no in‐gap state. There is one intense double‐lobed feature in the STM image [up pointing triangle in Figure 5a(ii)], which is caused by adsorption of CO at a Ti_5c_ site next nearest neighbor to the V_O_ (NNN‐Ti_5c_) and coupled to a polaron on the surface (S_0_ polaron). In Figure 5a(iii)–(v), on a more reduced surface (c_VO_ = 14.5%, θ_CO_ = 0.15 ML), all oxygen vacancies are occupied by CO with more CO at NNN‐Ti_5c_ observed. Increasing θ_CO_ to 0.57 ML (c_VO_ = 14.5%), the nc‐AFM image in Figure 5a(vi) clearly shows that CO avoids V_O_‐nearest‐neighbor sites, and prefers NNN‐Ti_5c_ sites in combination with the S_0_ polaron. As shown in Figure 5a(vii), double‐lobed features can be observed in the whole area. Corresponding DFT calculation suggested that when CO molecules are coupled with surface polarons, they will have lower adsorption energy, acquiring more polarized charges, as shown in the insets of Figure 3d–f. Hence, the adsorbed CO shows attractive coupling with polarons in the surface layer and repulsive interaction with polarons in the subsurface layer, respectively. Adsorption of CO at V_O_ or at NNN‐Ti_5c_ is very stable. As the V_O_ and polaron concentration increases, CO absorption at Ti_5c_ sites with no S_0_ polarons is less favorable. On the strongly reduced TiO_2_ surface, polarons are promoted to hop from S_1_ to S_0_ by the absorption of CO.

The interaction between CO and polarons in TiO_2_ reveals a mechanism of how adsorbate is affected by polarons. In addition to the effect on adsorption of reactants, the role of polarons in facilitating the loading of co‐catalyst such as single‐atom, has been reported. Existing studies have illustrated that single‐atom catalysts (SACs) can be effectively adsorbed on surface defect sites, thereby improving the stability of SACs and enhancing the efficiency and applicability of SACs.^[^ ^80^, ^81^, ^82^ ^]^ For example, Sombut et al. found that when a single metal atom is adsorbed on the defective rutile (110) surface at different positions, the valence state of SACs will change with the surface polarons.^[^ ^82^ ^]^

### 4.2 Photoexcitation

Photoexcitation is one of the determining steps in photocatalysis and photoelectrocatalysis, which is related to the range of light that catalyst can absorb and the efficiency of energy conversion. As polarons can introduce in‐gap bands, it is worthy to take this effect into consideration in the process of photoexcitation.^[^ ^83^, ^84^, ^85^ ^]^

The excitation of surface‐localized polaronic states has recently been discussed as an additional photocatalytic channel. Meanwhile, bulk polarons are also believed to contribute to photoexcitation, which had been studied in recent articles through the method of 2PPE by Tanner et al.^[^ ^27^ ^]^ As shown in Figure 5b(i), excitation scheme of bulk and surface polaron in rutile (110) was obtained by the combined results of DFT calculations and 2PPE spectra data. Both the initial and excited states of bulk polaron are 0.2 eV higher energy than that of surface polaron. 2PPE data showed a 3.54 eV excitation energy for both bulk and surface polarons, which confirms that the contribution arising from bulk polarons could not be ignored in photocatalysis.

Adsorbates were also believed to influence photoexcitation of polarons. Figure 5b(iii),(iv) show another work by Tanner et al. of how polaron‐adsorbate coupling affect photoexcitation of polaron in rutile (110).^[^ ^28^ ^]^ In this work, reduced rutile (110) sample (R110) was partially hydroxylated in UHV (Hp‐R110, ∼0.05 ML) and was sequentially exposed to gas‐phase formic and acetic acid (FA‐ and AA‐R110, ≈0.5 ML), during which 2PPE spectra was used to measure the resonance intensity (Figure 5b(iii)). Upon creation of FA‐ and AA‐R110, the dominant incoherent process is found to be ≈3 and 2 times larger, respectively (taken by peak area). Figure 5b(iv) shows the 2PPE spectra differences of different coverage of formate on TiO_2_ surface. With the increase of the coverage, two features marked as 1 and 2 appear in 2PPE spectra, which are believed to associate with the attraction to electrons at Ti_int_ by formate. Carboxylate adsorption could lead to polaron redistribution toward the surface, driven by the migration of Ti_int_, and may thus increase the density of polarons on surface, which can protect polarons against oxidation and enhance photoexcitation.

### 4.3 Overpotential of Reaction

The existence of polarons could be beneficial in some reaction steps in catalysis process. Catalytic reactions can usually be divided into several steps, where energy change occurs at every single step reaction namely the change of Gibbs free energy. In a work by Gono et al., they optimized the reaction path in the oxygen evolution reaction (OER) in rutile by hybrid DFT,^[^ ^79^ ^]^ as shown below:

(17) $$\begin{array}{c} {}*+H_{2}O\to {\mathrm{OH}}_{\mathrm{ads}}+H^{+}+e^{-} \end{array}$$

(18) $$\begin{array}{c} {\mathrm{OH}}_{\mathrm{ads}}\to O_{\mathrm{ads}}+H^{+}+e^{-} \end{array}$$

(19) $$\begin{array}{c} O_{\mathrm{ads}}+H_{2}O\to {\mathrm{OOH}}_{\mathrm{ads}}+H^{+}+e^{-} \end{array}$$

(20) $$\begin{array}{c} {\mathrm{OOH}}_{\mathrm{ads}}\to *+O_{2g}+H^{+}+e^{-} \end{array}$$

In this work, they suggested that the first step is the reaction‐determining step. The calculated free energies of the four steps with and without hole polarons on rutile surface is shown in Figure 5c. When hole polarons are introduced in this calculation, the overpotential of the first step was reduced, which could partially overcome the limitations in the linear scaling relationship in OER.

### 4.4 Mobility and Lifetime of Photogenerated Charge Carriers

Excess electrons or holes could also be injected into crystals by photoexcitation, which is a general process in photocatalysis and photoelectrocatalysis. In some photocatalysts, small polarons formed by trapped photoexcited carriers in crystal lattice. This localization of photoexcited carriers would affect charge transport in these materials because there is a significant difference in mobility between small polarons and free carriers. Haematite (α‐Fe_2_O_3_) is a promising photocatalyst for water splitting and artificial photosynthesis.^[^ ^86^ ^]^ However, the efficiency of existing haematite electrodes is far from their potential efficiency, which has been attributed to small polarons.^[^ ^22^, ^87^ ^]^ Carneiro et al. studied the localization of photoexcited electrons in haematite by transient extreme‐ultraviolet (XUV) spectroscopy.^[^ ^22^ ^]^ They suggest that majority of electrons and optical phonons combine and form small polarons within ≈2–3 ps after optical excitation, after which excited electrons recombine in a few hundred ps. The formation of small polarons would reduce charge carrier mobility and lifetime of haematite. It had also been pointed out in this work that by increasing excitation energy, hopping frequency and mobility of small polarons can be increased, which would suppress charge carrier recombination and extend lifetime of charge carriers.

In addition, temperature is also believed to affect mobility of small polarons and lifetime of photoexcited charge carriers. Zhang et al. investigated photoinduced small polarons in rutile at different temperatures using ab initio nonadiabatic molecular dynamic simulation.^[^ ^88^ ^]^ They pointed that, at 100 K and 300 K, the small polaron was mostly localized on a single Ti atom, and electron‐hole recombination occurred within several ns, which is comparable to that of free carriers. But at 700 K, small polarons were thermally excited to hop frequently, which increased the coupling between valence band maximum (VBM) and polaron states. Then electron‐hole recombination would be enhanced, leading that the time scale of recombination decreases to a few hundred ps. As small polarons have been widely found in many other transition metal oxides, studying the photoinduced polarons could bring a new understanding of photocatalytic behavior in these materials.

### 4.5 Surface Structure

Surface structure has great impact on physical and chemical properties of semiconductors. In catalysis, the effect of surface structure involves molecular adsorbing, separation/recombination of charge carriers, surface electronic properties, and surface reactive sites. Polarons had also been proved to have influence on surface structure, which may provide a new method in surface control.

Reticcioli et al. found that a (1 × 2) reconstruction can be caused by polarons on rutile (110). (1 × 1) structure and (1 × 2) reconstruction are identified by AFM and STM with the images shown in Figure 5d(i)–(v) and Figure 5d(ii)–(vi), respectively.^[^ ^29^ ^]^ The distribution of oxygen atoms and V_O_s was identified by nc‐AFM with an O tip termination on the unreconstructed surface with (1 × 1) structure and the reconstructed surface with (1×2) structure, as shown in Figure 5d(i),(ii), respectively. This (1 × 2) reconstruction, with Ti_2_O_3_ stoichiometry, can be regarded as Ti_2_O_4_ with 50% of V_O_s. Empty state and filled state STM on (1×1) surface show V_O_s and polarons originating from V_O_s, as shown in Figure 5d(iii),(v). On (1×2) reconstructed surface, filled state STM signal [Figure 5d(vi)] came from Ti^3+^ polaronic like states originating from Ti_2_O_3_ reconstruction. V_O_ concentration shows an evident difference [16.7% in (1 × 1), 50% in (1 × 2)] in these two structures, indicating the high concentration of polarons in (1 × 2) reconstructed surface. Calculation of polarons in rutile (110) surface gave that the Ti^3+^ polarons form in the subsurface sites at slightly reduced surface, corresponding to the (1 × 1) surface. But as the increase of V_O_s and polarons, the repulsion between negatively charged polarons results that Ti^3+^ polarons are pushed to the surface sites, and finally the (1×2) reconstruction with Ti_2_O_3_ stoichiometry forms.

## 5 Effect of Polarons on Catalyst Performance

### 5.1 TiO_2_

Metal oxide semiconductors and perovskites are often used in photocatalysis and photoelectrocatalysis. Polarons could form in these materials and play non‐negligible roles in catalysis. Here we list several examples and give a brief introduction about polarons effects in photocatalysis and photoelectrocatalysis.

**Figure 6:**
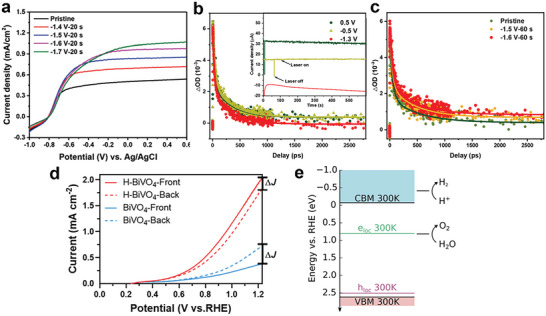
a) Photocurrent versus applied potential curves of TiO_2_ nanorod electrodes before and after electrochemical doping in 1 Z.R. and Z.S. contributed equally to this work. NaOH solution at potentials from −1.4 to −1.7 V for 20 s. Reproduced with permission.^[^ ^89^ ^]^ Copyright 2020, The Royal Society of Chemistry. b) Time profiles of the transient absorption of TiO_2_ nanorod electrodes at 700 nm under different applied potentials. The inset shows the I‐t curves for the TiO_2_ nanorod photoelectrodes obtained simultaneously under different applied potential during the transient absorption experiment. c) Time profiles of the transient absorption of TiO_2_ nanorod electrodes before and after electrochemical doping at −1.5 and −1.6 V for 60 s in 1 Z.R. and Z.S. contributed equally to this work. NaOH electrolyte at 700 nm after excitation by 350 nm femtosecond pulses under 0.5 V applied potential. Reproduced with permission.^[^ ^78^ ^]^ Copyright 2021, American Chemical Society. d) Current density‐voltage curves of pristine BiVO_4_ and H‐BiVO_4_‐(2.0:1.0, 180 °C) photoanodes measured under AM 1.5G simulated light illumination in 1 m KBi solution. Reproduced with permission.^[^ ^24^ ^]^ Copyright 2023, American Chemical Society. e) Alignment of the band edges of BiVO_4_ and polaron transition levels at 300 K with H^+^/H_2_ and O_2_/H_2_O redox levels at pH = 7. Reproduced with permission.^[^ ^25^ ^]^ Copyright 2018, American Chemical Society.

As a prototypical metal oxide photocatalyst, TiO_2_ has received long and wide concerns owing to its extraordinary catalytic performance and abundant characteristics. Small polarons could form in rutile TiO_2_ with the reduction of Ti sites from Ti^4+^ to Ti^3+^, and the reversible transformation of Ti^4+^ and Ti^3+^ is considered to be an effective pathway for massive electron‐transfer. Yang et al. proposed in their paper that, by electrochemically doping rutile TiO_2_ in NaOH solution under a negative bias, the density of polarons and PEC performance of rutile could be increased.^[^ ^89^ ^]^ As shown in **Figure** **6**a, TiO_2_ nanorod was used as electrode, the photocurrent‐potential curves were obtained in 1 m NaOH under 300 W Xe lamp irradiation. After electrochemical doping, with the increase of doping bias, the density of polaron in TiO_2_ increased, which led to the saturated photocurrent increased from 0.4 mA cm^−2^ in pristine TiO_2_ to 1.1 mA cm^−2^ in −1.7 V electrochemical doped TiO_2_.

In a later work by Zhu et al. In situ spectroelectrochemistry, femtosecond transient absorption spectroscopy (fs‐TAS) was used to study the effect of polarons on the lifetime of photogenerated electrons in TiO_2_.^[^ ^78^ ^]^ Figure 6b shows the TAS of TiO_2_ nanorod photoelectrodes, 350 nm pump pulse was used to excite electrons. The TA signal of −1.3 V curve rapidly dropped to around zero with time, which reflects the lifetime of photogenerated electrons, but the other two curves with more positive bias showed a longer lifetime of photogenerated electrons. Zhu et al. figured that at a large negative bias, polaron states were fully occupied. As a consequent, photogenerated electrons were difficult to be trapped into polaron states. With the bias became more positive, there were more unoccupied polaron states which could trap electrons and extend their lifetime. A comparison of TAS of different density of polaron, tuned by electrochemical doping TiO_2_ in a negative bias, is shown in Figure 6c. With the increase of polaron density, the lifetime of electrons extended. It is believed that, by trapping photogenerated electrons with polarons, the lifetime of electrons can be extended, which thus improves the PEC efficiency.

### 5.2 BiVO_4_

Ternary metal oxide semiconductor bismuth vanadate (m‐BiVO_4_) is a photoanode in photoelectrochemical water‐splitting that has received wide attentions.^[^ ^90^ ^]^ Small polarons could act as charge carriers of BiVO_4_ in photoelectrochemical water splitting.^[^ ^23^ ^]^ The transport of polarons, that is, hopping of polarons, could affect catalysis efficiency of BiVO_4_. Wu et al. proposed that in mildly hydrogenated BiVO_4_ (H‐BiVO_4_), the enhanced hopping of small polarons can increase its solar water oxidation efficiency.^[^ ^24^ ^]^ As shown in Figure 6d, at 1.23 V versus reversible hydrogen electrode (RHE), the photocurrent of H‐BiVO_4_ is 2.5 ± 0.02 mA cm^−2^, which is ≈5.3 times that of pristine BiVO_4_. In addition, photocurrent with front‐illumination of H‐BiVO_4_ is larger than back‐illumination, showing a different trend with pristine BiVO_4_. Correlation calculation for H‐BiVO_4_ indicates that, by mildly hydrogenating BiVO_4_, substitution hydrogen dopants occupy oxygen vacancies, which can decrease the hopping barrier of small polarons and promote the transport of polarons as charge carriers. Therefore, photoelectrochemical water‐splitting efficiency can be increased as nonradiative recombination loss of photoinduced electrons is suppressed. With the enhancement of small polaron hopping, photocurrent with front‐illumination has also been greatly improved.

Wiktor et al. had proposed that electron‐hole recombination through polaronic states would compete with the evolution of water‐splitting reaction.^[^ ^25^ ^]^ By combining thermodynamic integration method and hybrid functional molecular dynamics simulations, electron polaron and hole polaron in BiVO_4_ were studied. Electron polaron localizes at a V atom with the valence state of V turned to +4 from +5, and V‐O bond length increases. Hole polaron is mostly distributed between one Bi atom and eight O atoms with Bi‐O bond shortening. Electron polaron and hole polaron levels at 300 K were calculated, which are shown in Figure 6e together with band edge of BiVO_4_ and H_2_O redox level at pH = 7. For an ideal single‐absorber photoelectric water splitting catalyst, H^+^ reduction level is positioned below CBM and H_2_O oxidation level is positioned above VBM. However, in this calculation, they found that electron polaron level lies at 1.22 eV below H^+^ reduction level, causing water reduction difficult. Meanwhile, the electron polaron level is very close to H_2_O oxidation level, which means electron‐hole recombination could compete with the charge transfer to the electrolyte.

## 6 Summary

In this review, we summarized the basic physical models of polarons and recent progresses on the experimental characterization of polarons in materials. Abundant analysis techniques including microscopies and spectroscopies enable researchers to study polarons from various aspects. Specifically, STM has been used to directly observe polaron sites and atomically study the behaviors and surface‐effects. In situ spectroscopies make it possible to observe the changes of polarons in catalysis process. With the high time resolution techniques been invented, even the formation process of polaron can be obtained.

In addition, recent works of exploring the role of polarons in the photocatalysis and photoelectrocatalysis are reviewed. Those works demonstrated that polarons and their electronic states in photocatalysts and photoelectrodes could be key points in their properties. Surface polarons could interact with adsorbates, by studying and modulating the concentration and the distribution of polarons in catalysts, the effect of molecule adsorbing in catalysis could be further understood or even controlled. Surface reconstruction may also be caused by polarons, which could provide a method to modify surface active sites by controlling the formation of polarons. The electronic states introduced by polarons provide additional light absorption channel, thus expanding light absorption range and enhancing photocatalysis. The interconversion of depletion states and non‐depletion states of polarons is believed to provides a pathway in charge transport, which could promote charge separation and extend lifetime of charge carriers. In addition to polarons induced by defects or doping, in‐depth understanding of photogenerated intrinsic polarons also needs to be further explored in order to promote more efficient electron‐hole separation and their transport in catalysts, as exemplified by TiO_2_ and Fe_2_O_3_.

Although many studies have proposed the effects and corresponding theories of polarons from one or several aspects, more detailed mechanism of polarons in photocatalysis and photoelectrocatalysis still remains vague. More techniques and methods need to be carried out especially in real chemical and catalytic condition. In situ and high time resolution techniques are popular in catalytic studies and many of these techniques are also suitable in studying polarons in photocatalysis and photoelectrocatalysis. By performing in situ microscopies such as STM and nc‐AFM, surface structure, polaron distribution, molecule adsorption, and interaction before and after the catalysis process are expected to be observed. Considering the limit of scanning speed, microscopies are usually unable to observe the polaron evolution and detailed processes of catalysis, as the time scale of charge excitation and polaron formation is fs and the time scale of catalysis process is around ps to µs.^[^ ^91^ ^]^ Moreover, in situ and high time resolution spectroscopies may enable us to analyze and understand the role of polarons. In situ X‐ray diffraction analysis (XRD) and Extended x‐ray absorption fine structure (EXAFS) could obtain structure changes corresponding to polaron such as bond length and coordination. Photoelectron spectroscopies and absorption spectroscopies such as 2PPE, X‐ray photoelectron spectroscopy (XPS), and X‐ray absorption spectroscopy (XAS) can be used to recognize polaron states and valence changes. High time resolution or in situ Fourier‐transform infrared spectroscopy (FTIR), Raman, and EPR etc. are frequently‐used techniques in studying surface species and intermediates, and these techniques can contribute to the study of the role of polarons such as polarons’ effects on the intermediates and selectivity of catalysis. In addition, electrochemical test can also be used to identify polarons. For instance, photocurrent and Tafel slope could be used to test the catalytic efficiency and steps with and without the participation of polarons. Electrochemical Impedance Spectroscopy (EIS) may also be used to test the effect of polarons on carrier mobility. Furthermore, by developing advanced techniques and methods, and by combing various techniques especially in situ and high time resolution techniques, further and deeper understandings of polarons in photocatalysis and photoelectrocatalysis are expected to be obtained, which would facilitate the understanding of photocatalysis and photoelectrocatalysis and promote the design of more effective photocatalysts and photoelectrodes.

## Conflict of Interest

The authors declare no conflict of interest.
